# Is a preoperative pathogen detection a prerequisite before undergoing one-stage exchange for prosthetic joint infection of the hip?

**DOI:** 10.1007/s00402-022-04459-5

**Published:** 2022-05-04

**Authors:** Daniel Karczewski, Yannick Seutz, Christian Hipfl, Doruk Akgün, Octavian Andronic, Carsten Perka, Sebastian Hardt

**Affiliations:** 1 Center for Musculoskeletal Surgery, Department of Orthopaedic Surgery, Charité – Universitätsmedizin Berlin, Charitéplatz 1, 10117 Berlin, Germany; 2 Orthopedic Surgery, Balgrist University Hospital, Forchstrasse 340, CH-8008 Zurich, Switzerland

**Keywords:** One-stage exchange, PJI, Culture negative, Pathogen detection

## Abstract

**Background:**

A preoperative pathogen detection is considered a prerequisite before undergoing one-stage exchange for prosthetic joint infection (PJI) according to most guidelines. This study compares patients with and without preoperative pathogen detection undergoing one-stage exchange for PJI of the hip. The authors put up the hypothesis that a preoperative pathogen detection is no prerequisite in selected cases undergoing one-stage exchange.

**Methods:**

30 consecutive patients with PJI of the hip, treated with one-stage exchange, between 2011 and 2021, were retrospectively included. Mean age was 70 years and mean follow-up 2.1 ± 1.8 years. PJI was defined according to the European Bone and Joint Infection Society. One-stage exchange was performed in (1) chronic PJI longer than 4 weeks, (2) well-retained bone condition, (3) absence of multiple prior revisions for PJI (≤ 2) with absence of difficult-to-treat pathogens in the past, and (4) necessity/preference for early mobility due to comorbidities/age.

**Results:**

One-stage exchange was performed in 20 patients with and in 10 without a preoperative pathogen detection. Age (71 years, 68 years, *p* = 0.519), sex (50% and 30% males, *p* = 0.440), American Society of Anesthesiologists Score (2.2, 2.4, *p* = 0.502), and Charlson Comorbidity Index (3, 4, *p* = 0.530) did not differ among the two groups. No significant differences were noted concerning preoperative CRP (15 mg/l, 43 mg/l, *p* = 0.228), synovial cell count (15.990/nl, 5.308/nl, *p* = 0.887), radiological signs of loosening (55%, 50%, *p* = 0.999), and intraoperative histopathology. Except a higher rate of coagulase-negative staphylococci (70%, 20%, *p* = 0.019) in patients with a preoperative pathogen detection, no significant differences in pathogen spectrum were identified among groups. Revision for PJI recurrence was performed in one patient with an initial preoperative pathogen detection (3.3%). Additional revisions were performed for dislocation in two and postoperative hematoma in one patient. Revision rate for both septic and aseptic causes (*p* = 0.999), stay in hospital (16 and 15 days, *p* = 0.373) and modified Harris Hip Score (60, 71, *p* = 0.350) did not differ between groups.

**Conclusion:**

Patients with and without a preoperative pathogen detection did not show significant differences concerning baseline characteristics, clinical and functional outcomes at 2 years. An absent preoperative pathogen detection is no absolute contraindication for one-stage exchange in chronic PJI, if involving good bone quality and absence of multiple prior revisions.

## Introduction

Two-stage exchange is the most commonly used strategy in the treatment of chronic prosthetic joint infections (PJI), with most reported success rates ranging from 80 to 100% [1]. As an alternative, the one-stage exchange can be executed, which is being performed less frequently, although demonstrating comparable success rates compared to the two-stage exchange [2], while additionally offering potential advantages including the surgeon’s ability to combine antibiotic therapy and reimplantation in one sitting as well as introducing a less expensive, less time-consuming procedure, and earlier patient mobility [3]. Discussed reasons for the discrepancy between potential advantages and actual use of the one-stage exchange include fewer studies compared to the two-stage exchange resulting in less clinical experience, unawareness of the long-term outcome, as well as the fact that there are no universally accepted guidelines for the treatment of PJI, including a lack of universal indications for the one-stage exchange.

A final aspect potentially limiting the use of the one-stage exchange in clinical practice is the fact that a preoperative pathogen detection is considered a prerequisite according to current guidelines, including ENDO, University College London Hospital, and Infectious Diseases Society of America criteria [4–7]. In general, early pathogen identification and information via antibiogram about the sensitivity is important, allowing early targeted antibiotic therapy. Besides, certain difficult-to-treat-pathogens resistant to biofilm-active antimicrobials, as well as polymicrobial cases, and organisms not susceptible to antibiotics with high oral bioavailability should be treated with a two- or even three-stage exchange according to several studies [8–10]. However, excluding the one-stage exchange as a treatment option solely due to an absent preoperative pathogen detection should be critically reviewed for several reasons, including a lack of prospective RCT comparing one- and two-stage exchange, one stages’ potentially superiority concerning costs and functionality, as well as current research challenging present guidelines [11].

In this context, the authors put up the hypothesis that a preoperative pathogen detection is no prerequisite in selected cases undergoing one-stage exchange. This hypothesis will be evaluated by comparing baseline characteristics and outcome of patients with and without a preoperative pathogen detection undergoing one-stage exchange for PJI of the hip.

## Materials and methods

### Patient cohort

30 consecutive patients with chronic PJI of the hip, treated with one-stage exchange, between 2011 and 2021, were retrospectively included. 13 patients were males and 17 females. Mean age at one-stage exchange was 70 years, mean American Society of Anesthesiologists (ASA) score 2.2, and Charlson Comorbidity Index (CCI) 3. Coagulase-negative Staphylococci (CNS) (*n* = 16), Cutibacterium class (*n* = 8) and *Staphylococcus aureus* (*n* = 7) were the leading pathogens identified. The mean follow-up after one-stage exchange was 2.1 ± 1.8 years. 9 patients had a follow-up less than one, 20 less than 2 years.

### Definition of PJI

PJI has been set as confirmed if one of the following criteria according to the European Bone and Joint Infection Society (EBJIS) 2021 criteria was identified [12]: (1) sinus tract communicating with the prosthesis or joint, (2) < 3000/μl leukocytes or < 80% polymorphonuclear cells (PMN) in synovial fluid aspiration, (3) positive immunoassay or lateral-flow assay, (4) two or more positive samples with the same microorganism or sonication (> 50 colony forming units (CFU)/ml), (5) positive histopathology (≥ 5 neutrophils in ≥ 5 high power fields (HPF)). A PJI was considered likely, in case of (A) positive clinical features (loosening of prosthesis within 5 years of implantation or wound healing delay or recent fever or bacteremia or periprosthetic purulence) OR (B) increased CRP > 10 mg/l, COMBINED WITH (C) one additional criterion (joint aspiration > 1.500 leukocytes/µl or joint aspiration with > 65% PMN or positive culture in joint aspiration or single intraoperative culture or sonication (> 1 CFU/ml) or histopathology with ≥ 5 neutrophils in one single HPF or positive WBC scintigraphy). The EBJIS 2021 criteria used for definition of PJI and detailed evaluation in the present study are closely related to the criteria used before 2021 at our department, as defined by Trampuz et al. [13, 14]. All cases defined as PJI in the present study fulfilled at least one defining criteria according to both these historic and the current EBJIS 2021 criteria.

### One-stage exchange protocol

Treatment and diagnosis were performed in a university-based setting by an interdisciplinary team of orthopedic surgeons, microbiologist, pathologist, and infectiologists, exclusively specialized in prosthetic joint and implant-associated infections. One-stage exchange was performed in (1) chronic PJI with onset longer than 4 weeks, (2) well-retained bone and soft tissue condition (Paprosky IIB or lower grade at preoperative evaluation), (3) absence of multiple prior revisions for PJI (≤ 2) with absence of difficult-to-treat pathogens in the past, and (4) necessity/preference for early mobility due to comorbidities and age [15]. If at least one of the above criteria did not apply, a two-stage exchange was given higher priority.

Before one-stage exchange, diagnostical work-up and preparation of all instrumentation, including hardware to remove prosthesis and osteosynthesis material, antibiotic powder for cementation, and adequate mixing systems, were set up. One-stage exchange was then performed in a specialized operation room only covering septic cases. Prior to accessing intraarticular joint structures and opening the capsule, intraoperative aspiration was performed. After intraarticular access, at least five tissue samples were then obtained from the bone–implant interface for histological and microbiological analysis. All foreign material and hardware were removed, including restrictors, cement, and osteosynthesis material, and aggressive debridement and irrigation of the soft tissue and bone performed, including synovectomy and debridement of posterior and anterior hip capsule. A special focus was set on preservation of healthy tissue in the course of the radical debridement, differentiated from necrotic tissue by its capacity of bleeding [4]. Disinfection swaps were placed over the incision wound, and into the operative area, before new instrumentation and draping was set up, including rescrubbing, change of gown and gloves. Reimplantation was performed with antibiotic augmented cement providing additional local antimicrobial effects. No industrially pre-manufactures cement was used, but rather antibiotic powder based on the preoperative antibiogram. Patients with large bone defects were excluded in the present cohort. However, tantalum-based options were typically used in two-stage revisions involving large bone defects.

After surgery, all patients received a standardized regimen, including i.v. antibiotics without antibiofilm activity for the first 2 weeks, followed by 10 weeks of oral antibiotics with antibiofilm activity. Initially, empiric antibiotic therapy was used (Ampicillin/Sulbactam 3 × 3 g i.v., combined with Vancomycin 2 × 1 g i.v in septic patients or with multiple prior revisions). After intraoperative samples were taken, antibiotic therapy was continued according to the pathogen’s susceptibility [13, 14].

### Outcome

Outcome parameters included comparison between patients with and without a preoperative pathogen detection concerning baseline characteristics, definition of infection according to EBJIS criteria in patients without a preoperative pathogen detection, clinical (reinfection rate, antibiotic suppression therapy, aseptic revision rate), as well as functional outcome (non-surgical complications, modified Harris Hip Score, duration of hospital stay). Reinfection was defined as stated by the Delphi Consensus Criteria [16]: (1) wound healing delay with fistula, drainage, or recurrent infection caused by the same microbe, (2) surgical intervention for infection, and (3) PJI-related death. All outcome and patient characteristics were compared between patients with and without a preoperative pathogen detection.

### Statistics

Data were compared between groups using SPSS (IBM Corp., Armonk, New York). An independent-samples *t* test or Mann–Whitney U test were used for continuous variables, while the Fisher’s exact test was used for categorical variables. *α* value was set to *p* < 0.05, while a *p* value < 0.1 was interpreted as a trend to significance.

## Results

### Baseline and diagnostical characteristics

A preoperative pathogen detection was possible in 20 cases, while in 10 patients one-stage exchange was performed in the absence of a preoperatively identified pathogen (Table 1). 15 of the 20 cases with a preoperative pathogen detection underwent joint aspiration at our department prior to surgery, 12 of them yielding a positive culture, 10 a WBC count. The remaining eight culture positive cases were identified through open biopsy and debridement in one case each, as well as external diagnostical work-up in six patients. In addition, intraoperative joint aspiration was performed in 5 cases without and 11 with a preoperative pathogen detection, yielding positive cultures in 1 and 8 cases, respectively. Overall, 12 of 22 cases that underwent preoperative joint aspiration at our institution thus yielded a positive culture.

**Table 1:** Comparison of patients with and without a preoperative pathogen detection

	No preoperative pathogen detection	Preoperative pathogen detection	*P*
Patient characteristics			
Patients (*n*)	10	20	–
Males/females (*n*)	3/7	10/10	0.440
Age in years (mean ± SD)	68.9 ± 9.6	71.7 ± 11.9	0.519
ASA (mean ± SD)	2.4 ± 0.6	2.2 ± 0.6	0.502
CCI (mean ± SD)	4 ± 0.02	3 ± 0.01	0.530
Diagnostic characteristics			
Revision prior to current one-stage exchange (*n*)	3	10	0.440
Time from last revision to one-stage exchange in years (mean ± SD)	1.0 ± 1.6	4.2 ± 4.1	0.228
Prosthesis dislocation or loosening signs on preoperative X-ray (*n*)	5	11	0.999
Preoperative CRP (mean ± SD)	43.6 ± 50.6	15.0 ± 13.6	0.228
Preoperative joint aspiration (*n*)	7	10	0.440
Leukocytes/nl in joint aspiration (mean ± SD)	5.308 ± 3.256	15.990 ± 28.310	0.887
I Krenn and Morawietz (*n*)	2	4	0.999
II Krenn and Morawietz (*n*)	1	8	0.204
III Krenn and Morawietz (*n*)	0	3	0.532
IV Krenn and Morawietz (*n*)	4	4	0.384
Pathogen spectrum			
Coagulase-negative *Staphylococci* (*n*)	2	14	**0.019**
*Cutibacterium class* (*n*)	2	6	0.682
*Staphylococcus aureus* (*n*)	2	5	0.999
*Escherichia coli* (*n*)	0	2	0.540
*Enterococcus* spp. (*n*)	0	2	0.540
*Corynebacterium* spp. (*n*)	1	1	0.999
*Klebsiella pneumoniae* (*n*)	0	1	0.999
Culture negative (*n*)	4	0	0.087
Monomicrobial (*n*)	5	14	0.425
Polymicrobial (*n*)	1	6	0.372

Age (71 years, 68 years, *p* = 0.519), sex (50% and 30% males, *p* = 0.440), ASA (2.2, 2.4, *p* = 0.502), and CCI (3, 4, *p* = 0.530) did not differ among the two groups. In addition, no significant differences were noted concerning preoperative CRP (15 mg/l, 43 mg/l, *p* = 0.228), synovial cell count (15.990/nl, 5.308/nl, *p* = 0.887), radiological signs of loosening, and distribution of Krenn and Morawietz types I to IV [17]. Except a higher rate of coagulase-negative staphylococci (20%, 70%, *p* = 0.019) in patients with a preoperative pathogen detection no significant differences in pathogen spectrum were identified among groups.

### EBJIS criteria in patients without a preoperative pathogen detection

The diagnosis PJI (according to the EBJIS 2021 criteria) was confirmed via significantly increased rates of white blood cell count in joint aspiration (> 3.000/μl) prior to the operation in 5 of 10 patients without a preoperative pathogen detection (Table 2). 3 of the remaining five cases had the combination of an increased CRP (> 10 mg/l), a typical clinical component (early component loosening, secretion, pain), and no other infection focuses in the body. In all of these three cases, a pathogen detection was possible intraoperatively, confirming the diagnosis PJI according to the 2021 EBJIS criteria. In the remaining two of the ten cases without a preoperative pathogen detection, PJI was already likely according to the 2021 EBJIS criteria based on the preoperative condition (anamnesis, CRP, joint aspiration), and ultimately confirmed intraoperatively in one case via pathogen detection (Table 2).

**Table 2:** PJI characteristics of patients without a preoperative pathogen detection

Age, sex	CRP mg/l preoperatively	Preoperative joint aspiration	Characteristics	Intra-/postoperatively identified microbe	EBJIS 2021
70, female	17.4 mg/l	7.290/μl	Primary implantation 3 years ago	*Cutibacterium acnes*	PJI confirmed preoperatively
72, male	0.9 mg/l	5.590/μl	Primary implantation 8 years ago, prosthesis loosening	*Cutibacterium acnes*, *Staphylococcus hominis*, *Staphylococcus epidermidis*	PJI confirmed preoperatively
80, male	5.4 mg/l	5.498/μl	Primary implantation 4 years ago, prosthesis loosening	Culture negative	PJI confirmed preoperatively
64, female	56.1 mg/l	1.262/μl (71% PMN)	Primary implantation > 1 month ago, secretion and pain	*Staphylococcus aureus*	PJI likely preoperatively, PJI confirmed intraoperatively
76, female	106 mg/l	n.a	Primary implantation > 1 month ago, secretion and pain	*Staphylococcus aureus*	PJI confirmed intraoperatively
76, female	9.1 mg/l	2.695/μl	Primary implantation 5 years ago, prosthesis loosening	Culture negative	PJI likely preoperatively, PJI likely intraoperatively
46, females	0.6 mg/l	11.123/μl (PMN 82%)	Primary implantation 6 years ago, pain for more than one year, additional urosepsis	Culture negative	PJI confirmed preoperatively
67, female	146 mg/l	3.700/μl (PMN 60%)	Primary implantation 5 years ago, prosthesis loosening, HCC with metastasis	Culture negative	PJI confirmed preoperatively
71, male	75 mg/l	n.a	Primary implantation 1.5 month ago, pain, hip dislocation	*Corynebacterium tuberculostearicum*	PJI confirmed intraoperatively
64, female	20.4 mg/l	n.a	Primary implantation 8 years ago, secretion, pain	*Staphylococcus epidermidis*	PJI confirmed intraoperatively

Overall, PJI was confirmed in all patients with a preoperative pathogen detection, and in nine of ten patients without a preoperative detection, according to the EBJIS 2021 criteria. The only case without a confirmed PJI diagnosis was considered to be a likely PJI according to the current 2021 EBJIS definition. In addition, all 30 cases were confirmed PJI according to the definition used at our department before 2021 [14, 15]. Except a significantly higher rate of CNS in patients with a preoperative pathogen detection, no significant differences were noted between pathogen distributions among both groups (Table 2).

### Clinical outcome

Revision for PJI recurrence was performed in one patient, an 87-year-old female that underwent one-stage exchange 22 years after index surgery. Escherichia coli and methicillin-susceptible *Staphylococcus aureus* (MSSA) were identified prior to one-stage exchange. 3 years later, the patient represented to our department with signs of reinfection. A preoperative joint aspiration revealed MSSA, and the patient underwent repeated one-stage exchange. Cultivation of intraoperative samples revealed additional involvement of Enterococcus faecium and Escherichia coli. The patient was also prescribed on long-term suppression therapy. As one revision for PJI was performed, and no patient died of PJI, the reinfection rate following the Delphi Consensus criteria was 3.3% for all 30 patients. Antibiotic suppression therapy longer than 6 months was prescribed in one patient without and in two with a preoperative pathogen detection, not showing a significant difference among groups (Table 1).

Additional revisions for non-PJI-related causes were performed in two patients without (debridement for postoperative hematoma, dislocation with acetabular component exchange) and one patient with a preoperative pathogen detection (postoperative dislocation). Additional surgical perioperative complications included intraoperative trochanter major fracture treated with cerclages and plate osteosynthesis in a patient, and one closed reduction following dislocation.

### Functional outcome

Modified Harris Hip Score (60, 71, *p* = 0.350) and duration of stay in hospital after surgery (16 and 15 days) did not differ between patients with and without a preoperative pathogen detection (Table 3). One patient suffered a quadriceps paralysis most likely attributed to a femoral nerve palsy. No loosening was noted in the 26 unrevised cases at last radiographic follow-up.

**Table 3:** Outcome in patients with and without a preoperative pathogen detection

	No preoperative pathogen detection	Preoperative pathogen detection	*p*
Follow-up time in months (mean ± SD)	29.3 ± 30.3	23.6 ± 18.7	0.948
Reinfection (Delphi Consensus criteria) (*n*)	0	1	0.999
Aseptic revision (*n*)	1	2	0.999
Antibiotic suppression > 6 months (*n*)	1	2	0.999
Death (*n*)	0	0	–
Modified Harris Hip Score (mean ± SD)	71.8 ± 17.7	60.4 ± 24.4	0.350
Postoperative hospital stay in days (mean ± SD)	15.3 ± 10.3	16.5 ± 7.5	0.373

## Discussion

The study analyzed 30 patients with PJI that underwent one-stage exchange in a single university center. The cohort demonstrated a high percentage of cases without a preoperative pathogen detection (one in three) and was able to show that patients with and without a preoperative pathogen detection did not differ concerning their outcome, while possessing similar baseline characteristics.

While one-stage exchange offers several potential advantages, including costs, time spent in hospital, and functionality, it is still used less often than its two-stage exchange counterpart [1, 2, 11]. The prerequisite of identifying a pathogen at a preoperative status, according to current guidelines, was discussed as a potential cause for this discrepancy in the introduction of this manuscript. The prerequisite of a preoperative pathogen detection before undergoing one-stage exchange is based on the concept that certain difficult-to-treat-microbe’s such as Rifampicin-resistant Staphylococci, Ciprofloxacin-resistant Gram-negative bacteria, and Candida, as well as polymicrobial and culture-negative cases cannot efficiently be treated by a single-stage surgical procedure [18, 19]. This opinion is also expressed by the latest consensus meeting on PJI: “Relative contraindications to performing a one-stage exchange may include lack of identification of an organism preoperatively” (Lichstein et al. 2014) [20, 21].

However, in the authors’ opinion, this concept should be critically reviewed for several reasons.

First, no prospective, randomized, multicenter level of evidence I study in the field of one-stage exchange is present at this point. The first prospective multicenter studies comparing one- and two-stage exchange are expected 2022 and following years [22, 23], thus demonstrating that final conclusions cannot be drawn at this point.

Besides, several studies contrast existing concepts. In a 2021 study, published in the Bone and Joint Journal, van den Kieboom et al. compared culture-negative patients, 30 treated with 1-stage exchange, and 75 with 2-stage exchange, at a minimum follow-up of 1 year. At a mean follow-up of 4.2 years, the authors could not identify a significant difference between one- and two-stage exchange concerning reinfection rate (16% vs. 20%; *p* = 0.691) or 1-year mortality (3% vs. 4%; *p* 0.999) [11]. Similar to Kieboom et al.’s study, Ilchmann et al. reported of no reinfection following one-stage exchange in 39 hip PJI, including 6 patients without a preoperative pathogen detection [24]. These results are also confirmed by Lange et al. (15 preoperatively culture-negative cases with one reinfection; 41 with a preoperative pathogen detection and 4 reinfections) [25], and Bori et al. (8 preoperatively culture-negative cases in 24 1-stage exchanges, and 1 reinfection) [26].

The risk of identification of difficult-to-treat pathogens such as Candida in intraoperative samples of culture-negative cases is still considered a main argument against one-stage exchange in absence of a preoperative pathogen detection. However, in a study by Jenny et al., no reinfection was noted after a follow-up of 2 years in patients without a preoperative pathogen detection undergoing one-stage exchange, and subsequent identification of Candida in intraoperative samples [27]. On the contrary, Klatte et al. reported of ten patients treated with one-stage exchange despite having a preoperatively confirmed fungal PJI. At a mean follow-up of 7 years, only one fungal reinfection was noted [28].

Finally, in addition to reinfection rates, additional outcome parameters must be considered. While the success of the one-stage exchange is not finally evaluated compared to the two-stage exchange, some studies indicate that one-stage exchange might be superior concerning total time spent in the hospital, (perioperative) complication rate, mortality, costs, and functionality [9, 29, 30].

Based on the presented literature references and the results of the present study, we suggest the one-stage exchange as possible treatment strategy in the absence of a positive preoperative culture in patients with chronic PJI longer than 4 weeks, well-retained bone condition, absence of multiple prior revisions for PJI, absence of difficult-to-treat pathogens in the past, necessity/preference for early mobility due to comorbidities and age, and an otherwise competent immune system. Similar to that, Ji et al. proposed to use one-stage exchange exclusion criteria based on systematic and local extremity status such as immune system, tissue quality and prior revisions, while the pathogen detection was not used as a criterion. In their study, reinfection following 1-stage exchange was identified in 4 of 23 patients in culture-negative hip PJI, while 8 of 88 cases with an identified pathogen had an event of recurrent infection [31].

The remaining patient (age, sex, ASA, CCI) and diagnostical (white blood cells in joint aspiration, CRP, histopathology) characteristics of the present study do not differ significantly from results reported by other PJI studies [32]. This is also the case for the pathogen spectrum, with CNS, Cutibacterium class and Staphylococcus aureus representing the typical pathogens identified in hip PJI [32]. The authors put up the hypothesis that the significantly higher proportion of CNS in patients with a preoperative pathogen detection is rather a consequence of the four culture-negative cases in the group without a preoperative pathogen detection, than a preoperative pathogen group characteristic itself, as CNS resemble the typical pathogen spectrum.

Compared to other one-stage exchange studies, the reinfection rate in the present cohort was slightly lower (3.3%). In a meta-analysis by Kunutsor et al. [1], including a total of 38 1-stage exchange studies for PJI of the hip at a median follow-up of 35 month, the total reinfection rate was 8.2%, while it was 7.9% for the two-stage exchange [33]. The slightly lower reinfection rate in the present cohort might be the consequence of a shorter follow-up (2.1 vs. 2.9 years). While the rate of reinfection was low, non-PJI-related surgical complications following 1-stage exchange were moderate to high (16.6%, 5 of 30 patients). To the best of the authors’ knowledge, no systematic analysis has yet summarized the complication rates following one- or two-stage exchange for PJI. A study by Thiesen et al. (ENDO working group, 2021) identified medical complications in 30 out of 385 1-stage exchanges for PJI, thus demonstrating a significantly lower complication rate compared to the 2-stage exchange (9 of 44, OR 3.5, *p* < 0.01) [34]. The authors speculate that the complication rate might be associated with absolute numbers of an operation performed, and thus being lower in the ENDO clinic as a hospital specialized in the one-stage exchange. In contrast, our department has a stronger focus on two-stage exchanges with comparable numbers to the one-stage exchange performed in Hamburg. Similar to the present study, mortality was low in Thiesen et al.’s study (3 of 385 cases). However, while duration of hospital stay was 23.9 days in Thiesen et al.’s study, patients in the present study left the hospital 16.1 days after surgery [1].

Limitations of the present study did include its retrospective setting, a limited patient number and short follow-up (2.1 years). In addition, the inclusion of culture-negative cases was—together with increased CRP and anamnesis—based on the 2021 EBJIS joint aspiration criteria of more than 3000 cells [12]. While this cut-off has been shown specify and sensitivity values of 90% according to a 2018 meta-analysis [1], these results could not be confirmed by all studies [35]. PJI definition in these culture-negative cases are thus not necessarily fulfilled according to other definitions such as the Musculoskeletal Infection Society criteria, in which aspiration is considered a minor criterion only [36].

## Conclusion

One-stage exchange is demonstrating low reinfection rates (3.3%) at a short-term follow-up. Patients with and without a preoperative pathogen detection did not differ concerning their outcome and baseline characteristics. One-stage exchange in the absence of a positive preoperative culture might be considered in patients with chronic PJI, well-retained bone condition, absence of multiple prior revisions for PJI, and necessity for early mobility due to comorbidities and age.
